# Progress for *Journal of Functional Morphology and Kinesiology* in 2022

**DOI:** 10.3390/jfmk8010030

**Published:** 2023-02-24

**Authors:** Giuseppe Musumeci

**Affiliations:** 1 Department of Biomedical and Biotechnological Sciences, Anatomy, Histology and Movement Sciences Section, School of Medicine, University of Catania, Via S. Sofia 87, 95123 Catania, Italy; g.musumeci@unict.it; 2 Research Center on Motor Activities (CRAM), University of Catania, 95123 Catania, Italy; 3 Department of Biology, Sbarro Institute for Cancer Research and Molecular Medicine, College of Science and Technology, Temple University, Philadelphia, PA 19122, USA

## 1. Looking Back on 2022

The *Journal of Functional Morphology and Kinesiology* (JFMK, ISSN: 2411-5142), which was first released in March 2016, saw significant developments in 2022. This journal provides an advanced forum for research studies on functional morphology and kinesiology and the regulatory functions of movement. JFMK meets the growing demand for high-quality, peer-reviewed international journals, offering easy access, high publicity via open access, the Digital Object Identifier (DOI), ORCID, and CrossRef to all researchers. We are indexed in Scopus (Elsevier’s abstract and citation database), PubMed, PMC, DOAJ (Directory of Open Access Journals), Scilit (a comprehensive, open-access scholarly database developed and maintained by MDPI), Google Scholar, World Health Organization Hinari, Food Science and Technology Abstracts (FSTA), IFIS, and Norwegian Register for Scientific Journals, Series and Publishers (NSD). Our full texts are archived in CLOCKSS (Digital Archive), e-Helvetica (Swiss National Library Digital Archive), and J-Gate (Informatics India).

In 2022, JFMK was assessed by SCImago Journal Rank as having a 2.7 CiteScore and reached Q2-quartiles in the following research fields: Anatomy, and Physical Therapy, Sports Therapy and Rehabilitation.

In 2023, the CiteScoreTracker result for our journal in Scopus is 3.6, which is higher than in the previous year, which demonstrates the citation growth of our journal. In October 2022, we applied to be indexed in the Web of Science.

JFMK is a member of the Committee on Publication Ethics (COPE). To verify the originality of content submitted to our journals, we still use iThenticate to check submissions against previous publications. MDPI works with Publons to provide reviewers with credit for their work and with MDPI Scitations Alert to provide our authors information on new publications in their research field.

The journal publishes articles focusing on molecular, cellular, tissue, system, and the whole-body response to a broad definition of physical activities. Furthermore, the journal provides an advanced forum for the analysis of the structure, function, development, and evolution of the cells and tissues of the musculoskeletal system and associated clinical disorders. We are proud to let you know that, thanks to your continuous support, the *Journal of Functional Morphology and Kinesiology* has continued to grow on functional morphology and kinesiology research dealing with the analysis of structure, function, development, and evolution of cells and tissues of the musculoskeletal system and the whole body. It is my pleasure to confirm the progress recorded in recent years [1,2,3,4,5], as stated in our statistics https://www.mdpi.com/journal/jfmk/stats (accessed on 15 January 2023).

Indeed, the number of published manuscripts has jumped from 98, in the 2021 volume, to 112, in the 2022 volume, and we rejected 35% of the contributions to maintain the high standards of our journal. The *Journal of Functional Morphology and Kinesiology* receives more manuscripts than it is able to publish, and the decision as to which papers are accepted or rejected is a difficult one. The decision is based on several factors, including originality, experimental design, scientific quality, data interpretation, clarity, and the quality of the written English, in order to uphold the high standards we have set for our journal.

In 2022, different Special Issues were activated thanks to the huge support of our editors. They include the following: “Motor Competence, Physical Activity and Health 2022”, edited by Prof. Dr. Vítor P. Lopes and Prof. Dr. Luis Paulo Rodrigues [6]; “Motivational Factors Influencing Performance in Sport and Exercise”, edited by Dr. Christopher Ballmann [7]; “3D Analysis of Human Movement, Sport, and Health Promotion”, edited by Dr. Luca Petrigna [8]; “Applied Sport Physiology and Performance—3rd Edition”, edited by Dr. William Guyton Hornsby III [9]; “Role of Exercises in Musculoskeletal Disorders—5th Edition”, edited by Prof. Dr. Giuseppe Musumeci [10]; “Sports Performance Analysis”, edited by Dr. Javier García-Rubio [11]; “Research on Sports Nutrition: Body Composition and Performance 3.0”, edited by Dr. Jose Antonio [12]; “Health and Performance through Sports at All Ages 2.0”, edited by Dr. Gianpiero Greco [13]; “Movement and Balance”, edited by Prof. Dr. Olivier Hue [14]; “Efficiency in Kinesiology: Innovative Approaches in Enhancing Motor Skills for Athletic Performance”, edited by Dr. Diego Minciacchi [15]; “Health Promotion in Children and Adolescents through Sport and Physical Activities—4th Edition”, edited by Dr. Antonino Bianco [16].

All articles published in the *Journal of Functional Morphology and Kinesiology* will be published in full open access in order to provide free access to readers, and to cover the costs of peer review, copyediting, typesetting, long-term archiving, and journal management, an article processing charge (APC) of 1600 CHF (Swiss Francs) will be applied to papers accepted after peer review.

In 2022, we added new keywords related to our journal as follows: Exercise and Physical Health; Sports Psychology and Cognitive Functioning; Muscle Structure and Musculoskeletal Disorders; Anatomy and Kinesiology; Adapted Physical Activity for Health Promotion; Rehabilitation and Rheumatology; Sports Medicine, Injury Prevention and Treatment; Strength and Power; Nutrition and Body Composition; Physical Activity and Neurodegeneration; Postural Control and Balance; Resistance Training; Sport Physiology and Performance; Athlete Monitoring and Management; Team Sports and Technology.

## 2. Looking Forward to 2023

In 2023, we shall continue our efforts to improve the journal through further growth and increased visibility.

In order to achieve this target and lay a strong foundation for publications in 2023, and in our application for indexing, we have made the following plans:

- Follow up the planned papers from editorial board members;
- Contact international conferences recommended by the Editor-in-Chief or by editorial board members and try to establish media partnerships with them to make JFMK increasingly well known among scholars;
- Communicate with editorial board members more frequently and seek their generous and valuable input and expertise for journal development;
- Post high-quality papers through social media (e.g., LinkedIn, Twitter, and Facebook) and increase online readership;
- Reduce the processing time of each submitted manuscript;
- Take steps to have publications indexed by the Emerging Sources Citation Index (Web of Science), by EMBASE (Elsevier) and by Web of Science—Clarivate;
- Improve the Citescore in the SCImago Journal Rank in the kinesiology-related sections such as Anatomy, Histology, Orthopedics and Sports Medicine, Physical Therapy, Sports Therapy, and Rehabilitation;
- Achieve the First Impact Factor status, as released by Clarivate Analytics;
- Accomplish, for our authors, the JFMK Best Paper Award and the JFMK Travel Grant Award;
- Garner, for the sake of journal promotion, support from sponsors for our editors to participate in, and disseminate our journal to, international conferences.

Since 2021, MDPI has included the accepting Academic Editor’s name on published articles, where they have accepted that manuscript after full peer review. This supports greater transparency for the readership, demonstrates the care that our Academic Editors take in making decisions, and offers full acknowledgement of the effort put in when making expert judgements about the suitability of a manuscript for publication. We strongly believe that this will also support the rigorous and robust quality of our peer-review process.

We hope that you share our enthusiasm for this journal, and we look forward to working with you to make JFMK a leader in its field. Your contributions are vital for the success of this new journal. We look forward to receiving your contributions (papers, reviews, etc.), and proposals for Special Issues are always welcome.

I have personally found this to be quite a challenge, one not helped by the COVID-19 pandemic, but one due in large part to the special position that JFMK is trying to navigate in the highly competitive publishing landscape. I wish you a healthy and prosperous new year and look forward to continuing to expand the reach and impact of the journal with your help next year.

I take also this opportunity to warmly thank, for their confidence, the following: our authors, readers, and reviewers, as well as our editorial advisors, eminent scientists in these fields, who, with their experience and important suggestions, guide us in this great enterprise; our excellent editorial board members, whose depth of experience covers a very broad spectrum on different disciplines related to morphology and kinesiology arenas; the managing editor, Ms. Molly Lu, for her huge support, the publishing manager, Dr. Peter Ribar, and the other members of the Editorial office, who day after day, thanks to their valuable contributions, ensure the growth of this journal; and finally, all members of our teams in Basel, Barcelona, Beijing, Belgrade, Romania, Tokyo, and Wuhan, as well as our sponsors.

## Data Availability

Not applicable.
